# Supervised Immediate Postoperative Mobilization After Elective Colorectal Surgery: A Feasibility Study

**DOI:** 10.1007/s00268-021-06347-2

**Published:** 2021-10-19

**Authors:** Rose-Marie W. Thörn, Jan Stepniewski, Hans Hjelmqvist, Anette Forsberg, Rebecca Ahlstrand, Olle Ljungqvist

**Affiliations:** 1 Department of Physiotherapy, Örebro University Hospital, Örebro, Sweden; 2 Department of Anesthesiology and Intensive Care, Örebro University Hospital, Örebro, Sweden; 3 Department of Anesthesiology and Intensive Care, Faculty of Medicine and Health, Örebro University, Örebro, Sweden; 4 Department of Surgery, Faculty of Medicine and Health, Örebro University, Örebro, Sweden

## Abstract

**Background:**

Early mobilization is a significant part of the ERAS^®^ Society guidelines, in which patients are recommended to spend 2 h out of bed on the day of surgery. However, it is not yet known how early patients can safely be mobilized after completion of colorectal surgery. The aim of this study was to evaluate the feasibility, and safety of providing almost immediate structured supervised mobilization starting 30 min post-surgery at the postoperative anesthesia care unit (PACU), and to describe reactions to this approach.

**Methods:**

This feasibility study includes 42 patients aged ≥18 years who received elective colorectal surgery at Örebro University Hospital. They underwent a structured mobilization performed by a specialized physiotherapist using a modified Surgical ICU Optimal Mobilization Score (SOMS). SOMS determines the level of mobilization at four levels from no activity to ambulating. Mobilization was considered successful at SOMS ≥ 2, corresponding to sitting on the edge of the bed as a proxy of sitting in a chair due to lack of space.

**Results:**

In all, 71% (*n* = 30) of the patients reached their highest level of mobilization between the second and third hour of arrival in the PACU. Before discharge to the ward, 43% (*n* = 18) could stand at the edge of the bed and 38% (*n* = 16) could ambulate. Symptoms that delayed advancement of mobilization were pain, somnolence, hypotension, nausea, and patient refusal. No serious adverse events occurred.

**Conclusions:**

Supervised mobilization is feasible and can safely be initiated in the immediate postoperative care after colorectal surgery.

*Trial registration Clinical trials.gov identifier*: NTC03357497.

## Introduction

Early postoperative mobilization is a well-established part of the multimodal evidence-based Enhanced Recovery After Surgery (ERAS) care pathway for surgical patients (http://www.erassociety.org/) [1]. Postoperative mobilization activities such as sitting, standing, and walking are key factors to minimize complications and reduce the length of hospital stay [2–4]. The most recent ERAS^®^ Society guidelines for colorectal surgery [1] recommend that patients should be mobilized out of bed on the day of surgery, at least by sitting in a chair for 2 h, and for the rest of the stay in hospital aim to be out of bed for at least 6 h daily. Performing mobilization at the postoperative anesthesia care unit (PACU) can be perceived as risky, resource-intensive, and with barriers that can be perceived as to high for patients [5, 6]. While early mobilization is advocated to prevent postoperative complications [7–9], there is lack of evidence how early after surgery the mobilization can be safely initiated and has not been investigated after colorectal surgery. The aim of this study was to evaluate the feasibility, and safety of providing immediate structured supervised mobilization to elective colorectal surgical patients in the PACU starting 30 min post-surgery, and to describe reactions to this approach.

## Material and methods

### Design

This study has its focus on the very immediate reactions to mobilization initiated as soon as possible at the PACU. The current report is part of a larger project to investigate the effects of this immediate intervention as opposed to the early mobilization initiated later on at the ward as suggested in ERAS programs. The main study is a randomized trial that is registered in Clinical Trials (NCT 03357497) where the effects of these two models of mobilization are compared with regard to different elements of recovery at different stages. In the current report, the effects and reactions to immediate mobilization are captured and discussed in detail.

Data were initially collected in a pilot study where 17 consecutive patients operated for elective colorectal surgery were subjected to immediate mobilization in December 2016 and January 2017. The pilot study revealed that immediate mobilization seemed feasible, since no safety issues could be identified. To broaden the material for the question raised in this study and to capture more variation in reactions, we also included the first 25 participants in the RCT randomized to the intervention group. No specific calculation was made to this sample size, but it felt to be reasonable in order to detect variations in how the patients were coping.

The inclusion criteria were age ≥18 years and planned for elective major open or laparoscopic colorectal surgery at Örebro University Hospital. Patients were excluded if they were unable to understand written information or were unable to walk preoperatively**.** The setting was the PACU of the hospital, a unit with 13 recovery beds in an open floor environment.

Written consent was obtained from each participant. The study was approved by the Regional Ethics Board of Uppsala, Sweden (ref: 2017/223).

## Definitions

### ERAS protocol

All patients were admitted on the day before surgery and received standard care according to the standardized ERAS protocol (Table 1). The same standard preoperative information was given to all patients by the physiotherapist (Table 1). All patients were also informed that they would be subjected to immediate mobilization, at the PACU or at the ward.

**Table 1:** The ERAS protocol for elective colorectal surgery: preadmission, preoperative, intraoperative, and postoperative procedures

*Preadmission*	
Preoperative counseling and patient education	Standardized oral and written information 2 weeks before surgery
Nutritional assessment	Evaluation for malnutrition (NRS 2002). If a need for dietary supplements is assessed, the patient receives a starter pack of 12 nutritional drinks (Fresubin^®^ Protein Energy, SWE, 200 ml)
Alcohol and tobacco	Tobacco and ethanol usage cessation before surgery
Admission	Admission in the afternoon on the day before surgery
Information from physiotherapist	Standardized information about the aim to achieve postoperative daily mobilization goals (out of bed on the day of surgery: at least by sitting in a chair for 2 h and for the rest of the stay in hospital: at least 6 h out of bed daily) and instructions to perform a set of ten consecutive deep breaths three with a 30–60 pause between each set at every hour with positive expiratory pressure device (PEP valve system 22, Rium Medical AB, Åkersberga, Sweden) at a mid-expiratory pressure of 10–15 cm H_2_O
*Preoperative*	
Preoperative fasting	Food permitted until midnight; clear fluids until 2 h before surgery
Carbohydrate treatment	Preoperative carbohydrate (preOp^®^Nutricia, NL, 400 ml) no later than 6 a.m. or 2 h before surgery
Bowel preparation	No routine bowel preparation
Preemptive analgesia	Paracetamol 1 g po. 1 h before surgery
Antibiotic prophylaxis	Sulfometaxazol + trimetoprim 160/800 mg
Thrombosis prophylaxis	Dalteparin 5000IE before 7 p.m. on the day before surgery
*Intraoperative*	
Anesthesia and pain management	
Multimodal analgesia	Parecoxib 40 mg iv. clonidine 30–45 µg iv, morphine 2–10 mg iv. and repeat dose paracetamol 1 g iv. if long surgery
Epidural anesthesia	In laparotomy cases, EDA with bupivacaine 5 mg/ml + adrenalin 5 µg/ml, continuous infusion or bolus. No EDA in laparoscopic cases
Anesthesia induction	Fentanyl 2 µg/kg, propofol 2 mg/kg
Anesthesia maintenance	Sevoflurane MAC 0.7–1.2 and intermittent fentanyl 1 µg/kg
PONV prophylaxis	Betamethasone 4 mg iv., ondansetron 4 mg iv
Fluid treatment	Intraoperative crystalloid Ringer-Acetate infusion 4–6 ml/kg/h
Patient warming strategy	Warmed infusions and hot air blanket
Drain and line management	No routine postoperative nasogastric tube. No routine abdominal drains
Urinary catheter	Inserted in the operating room
*Postoperative*	
Multimodal	Parecoxib 40 mg iv, clonidine 30–45 µg iv. paracetamol 1 g iv. If inadequate, titrated doses morphine 2–5 mg iv
Epidural	If EDA is present, a shift to PCEA and change of anesthetic to ropivacaine 2 mg/ml + sufentanil 1 µg/ml
PONV treatment	Betamethasone 4–8 mg iv., ondansetron 4 mg iv., droperidol 0.625–1.25 mg iv., metoklopramid 10 mg iv
Postoperative fluids	Glucose 2.5% 500 ml until next day if no oral intake and/or prolonged stay at PACU
Nutrition and fluids	Clear fluids offered at PACU for intake followed by nutritional drink 300 kcal
Discharge criteria from PACU	Respiration: unlabored breathing pattern with respiratory rate between 9 and 20; SaPO_2_ > 95% or preoperative value. Restored coughing and swallowing function
	Circulation: heart rate >45 and <110. Systolic blood pressure >90 and diastolic blood pressure <199. No new arrhythmia. No signs of myocardial ischemia. No active bleeding. Temperature > 36 °C. Pain: NRS < 4
	Neurology: Alert or preoperative status
	National Early Warning Score < 4

### Anesthetic regime and immediate postoperative care

Patients received standard anesthesia and analgesia care for colorectal surgery at our hospital (Table 1) including intraoperative prophylaxis for postoperative nausea and vomiting (PONV).

At the PACU standard patient monitoring was employed for all patients for at least an hour and thereafter until they met the discharge criteria (Table 1).

### Intervention

A mobilization protocol based on the Surgical ICU Optimal Mobilization Score (SOMS) [10] was used to structure and evaluate the mobilization intervention. This score describes mobilization from none (0) to ambulating (4). In the present study, it was modified to describe mobilization of surgical patients in the PACU (Table 2). A specialized physiotherapist with 30 years of experience working with patients in intensive care and with major colorectal surgery was in charge of the mobilization intervention for all the participants. The physiotherapist alone performed all mobilization in bed and until sitting on the bedside. Any mobilization beyond this level was performed with the assistance of a nursing assistant.

**Table 2:** Modified Surgical ICU Optimal Mobilization Score (SOMS), safety criteria, and barriers to mobilization

SOMS	Level 0 *No activity*	Level 1 *In-bed activity*	Level 2 *Sitting*	Level 3 *Standing*	Level 4 *Ambulating*
Definition	Mobilization was not possible	*(Modified definition from original)*^*a*^ Head end raised about 30 degrees AND active lower leg circulation exercise 20 reps,1 set	Sitting at the bedside with/without support	Standing twice with/without support of walking aid and assistance of two persons, and taking steps in-place	Ambulating 5–10 m with walking aid and assistance of two persons, one on each side of the patient
Safety criteria for advancing active mobilization^11^	Percutaneous oxygen saturation ≥90% Adequate blood pressure, fluid volume, and adequate pain control Postoperative vasopressor was not an absolute contraindication to mobilization, but the appropriateness of mobilization was discussed with the clinical team including the anesthesiologist If unsuccessful in achieving hemodynamic stabilization (abnormal electrocardiography, systolic blood pressure <90 mmHg) or pain control, or there are signs of motor blockage due to epidural analgesia, do not advance				

^a^The original definition of SOMS level 1 was PROM (passive range of motion)^10^. In this study, we modified the definition of SOMS Level 1 to in-bed activity

Mobilization started 30 min after arrival in the PACU, after receiving a report from the PACU nurse and completion of assessment according to the safety criteria for advancing mobilization (Table 2) [11]. The patient was then mobilized at 30 min intervals with 30 min of rest between attempts during the hours 1, 2, 3, and 4 of arrival to the PACU. At each mobilization attempt, the patient was advanced as far as they were able to along the stepwise SOMS mobilization schedule while adhering to safety criteria [10, 11]. This continued until the patient was discharged to the ward, or for a maximum of 4 h. Since the patient was bedridden at the start of each mobilization, each attempt started with activity in bed (level 1). If physiologically stable, the patient was mobilized to sit at the bedside (level 2). This was used as proxy for being mobilized to sitting in chair due to lack of space for chairs. From there, the patient was mobilized to the standing position and taking steps in place (level 3). If the patient still remained physiologically stable, they were asked to ambulate 5–10 m (level 4). If the mobilization was interrupted due to symptoms of unsafe mobilization, the patient was brought back to bed to rest and a new mobilization attempt was performed starting at level 1 after a 30-min break, regardless of how many levels the patient had completed during the previous rounds. If the patient reached SOMS level 4 and still remained in the PACU, a 60-min pause was allowed before mobilization began over again at level 1. In addition to the above, the patients performed breathing exercises during each mobilization attempt to the above [12–14].

### Data collection

Baseline demographics, perioperative data, and surgical data (Table 3) were extracted from the patient’s medical record and from a prospectively maintained database, the ERAS^®^ Interactive Audit System (EIAS) [15]. Analgesia and pain were estimated using the numeric rating scale (NRS) of 0–10 [16] before and after each mobilization session [17]. Opioid consumption, PONV, and blood pressure management were also recorded.

**Table 3:** Patients’ demographic, perioperative, and surgical data (*n* = 42)

Female sex	24 (57)
Age in years	69 (62–76)
*ASA group*	
1	23 (55)
2	13 (31)
3	5 (12)
Unknown	1 (2)
*WHO score*	
0	37 (88)
1	5 (12)
Diabetes on medication	8 (19)
BMI^a^	25.5 (23.1–29.1)
Current smoker	1 (2)
Alcohol usage	1 (2)
*Preoperative nutritional status*^*b*^	
Normal status	24 (57)
Risk of malnutrition	5 (12)
No, not assessed	1 (2)
*Presurgical therapy*	
Chemotherapy	2 (5)
Radiotherapy	10 (24)
Previous abdominal surgery	27 (64)
Previous PONV^c^	21 (50)
*Type of surgery*	
Abdominoperineal resection	6 (14)
Anterior resection of rectum	3 (7)
Right hemicolectomy	9 (21)
Left hemicolectomy	3 (7)
Other stoma procedures	8 (19)
Sigmoid resection	8 (19)
Total colectomy	4 (9)
Exploratory laparotomy	1 (2)
*Surgical approach*	
Open surgery	25 (60)
Standard laparoscopic	4 (9)
Robotic	11 (26)
Approach through existing stoma	2 (5)
Time of surgery, min^d^	174 (137–215)
Time anesthesia, min^e^	208 (191–269)
Total IV volume of fluids intraoperative ml^f^	1000 (525–1275)
Core body temperature at the end of operation^g^	35.95 (35.6–36.4)
Intraoperative blood loss, ml^h^	50 (0–100)

Values are reported as median (IQR) for ordinal or continuous variables and number (percentage) for categorical variables

ASA = American Society of Anesthesiologists (1 = normal healthy patient, 2 = mild systemic disease, 3 = severe systemic disease); WHO score = World Health Organization scale performance status (0 = normal, 1 = restricted in strenuous activity); PONV = postoperative nausea and vomiting; BMI = body mass index

^a^missing = 5; ^b^missing = 12; ^c^unknown = 7; ^d^missing = 1; ^e^missing = 15; ^f^missing = 1; ^g^missing = 16; ^h^missing = 2

Successful mobilization was defined as the patient being mobilized to sitting on the edge of the bed (as a proxy for being mobilized to sitting in a chair), standing, or ambulating (SOMS levels 2–4, respectively). Sitting on the edge instead of sitting on the chair as recommended by ERAS protocol was a compromise due to limitations in the PACU. Specific safety criteria (Table 2) were followed while advancing mobilization. Barriers hindering immediate advancement of mobilization and adverse events that occurred during the mobilization were recorded. Perioperative compliance with the ERAS colorectal protocol was recorded [18].

### Statistical analysis

All data were analyzed with descriptive statistics using version 25 of IBM SPSS Statistics. Descriptive statistics for categorical variables were reported as frequency (percentage), while continuous or ordinal variables were presented as median (range or interquartile range).

## Results

A total of 42 patients were included in the study. Demographic, perioperative, and surgical data are shown in Table 3. Two patients required conversion to an open surgery. Six patients had surgery lasting more than 240 min (rectal resection *n* = 4, colonic resection *n* = 1, other stoma procedures *n* = 1).

**Fig. 1:**
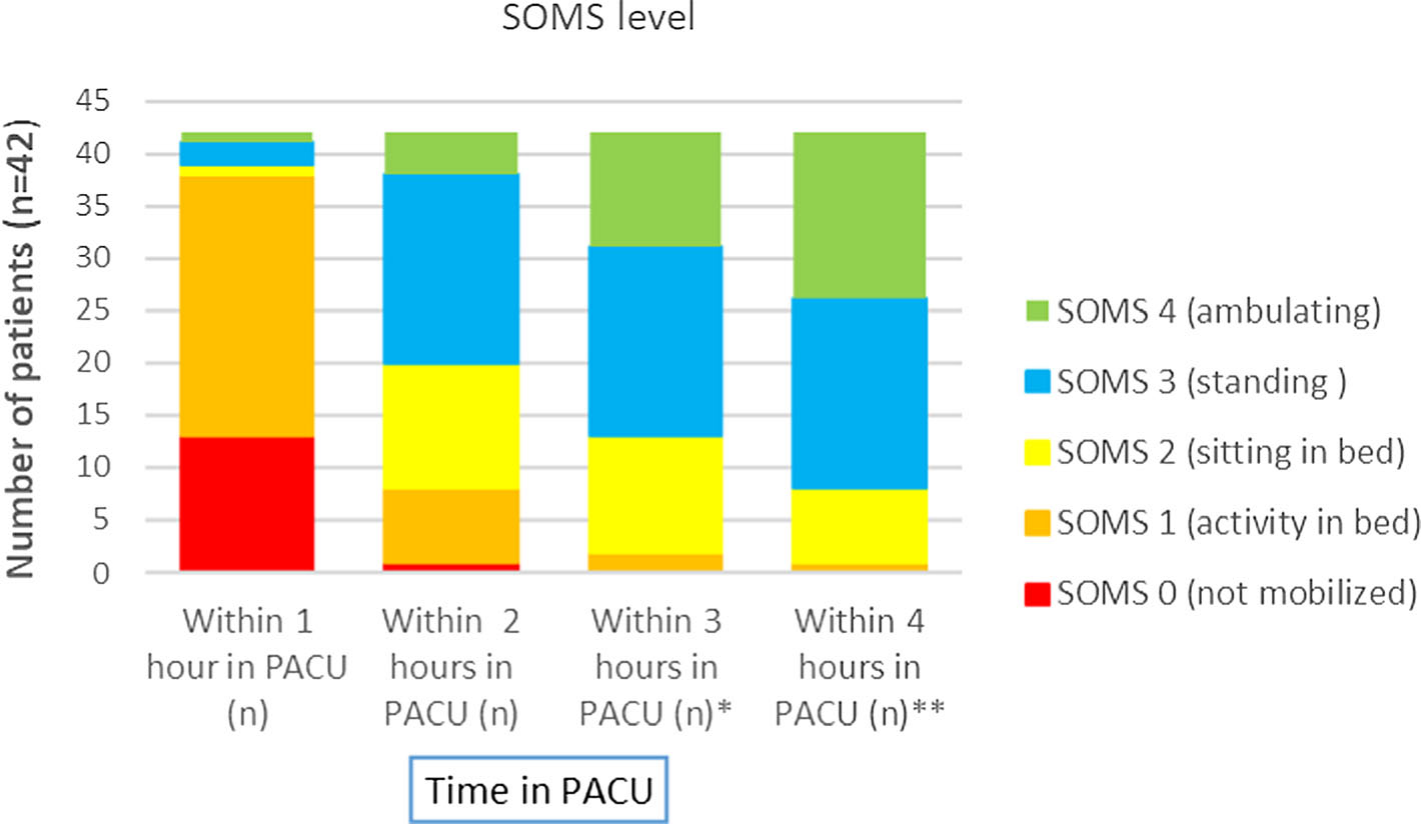
SOMS level achievement per time period in the PACU (*n* = 42). *Discharge from PACU (*n* = 11) ** discharge from PACU (*n* = 29)

All but one patient could be successfully mobilized at least to sitting, and 34 (81%) was mobilized out of bed (standing/ambulating) (Table 4). Thirty patients (71%) reached their highest level of mobilization being sitting to ambulating (SOMS 2–4) between the second and third hour of arrival in the PACU. Four patients (10%) were able to be mobilized to sitting, standing, and ambulating in the first hour after arrival. Details are presented in Table 4 and Fig. 1. No severe adverse event occurred during mobilization of any of the patients. Somnolence (*n* = 28) and pain (*n* = 21) were the main barriers that hindered immediate advancement of mobilization.

**Table 4:** Mobilization of patients in the PACU (*n* = 42)

Surgical approach	Highest achieved mobilization level	Within 1 h in PACU (*n*)	Within 2 h in PACU (*n*)	Within 3 h in PACU (*n*)	Within 4 h in PACU (*n*)	Total patient (*n*)
Open (*n* = 25)	SOMS 1 (activity in bed)			1		1
	SOMS 2 (sitting in bed)	1	2	1		4
	SOMS 3 (standing)		7	2	2	11
	SOMS 4 (ambulating)	1	1	5	2	9
Laparoscopic (*n* = 4)	SOMS 2 (sitting in bed)		2			2
	SOMS 3 (standing)		1			1
	SOMS 4 (ambulating)				1	1
Robotic (*n* = 11)	SOMS 3 (standing)	1	3		1	5
	SOMS 4 (ambulating)		2	2	2	6
Approach through existing stoma (* n* = 2)	SOMS 2 (sitting in bed)		1			1
	SOMS 3 (standing)			1		1

Details of postoperative pain and perioperative opiate consumption are described in Table 5. The three patients requiring vasopressor treatment for hypotension when arriving at the PACU reached a highest SOMS level of 2 (*n* = 1) and 3–4 (*n* = 2), respectively. For all three patients, the vasopressor treatment could be discontinued before discharge. PONV was treated with ondansetron (Hameln, Germany); 20 patients received one dose, six patients received two doses, and three patients received three doses. One patient received a combination of ondansetron and betamethasone postoperatively.

**Table 5:** Perioperative pain management (*n* = 42)

Epidural	PCA	Perioperative opiates				Postoperative opiates			NRS^a^ before/after mobilization			
*n* (%)	*n* (%)	(*n* = 36) Fentanyl, μg	(*n* = 7) Remifentanil, μg	(*n* = 24) Morphine, mg	(*n* = 1) Ketobemidone, mg	(*n* = 20) Fentanyl, μg	(*n* = 20) Morphine, mg	(*n* = 2) Ketobemidone, mg	(*n* = 19) within 1 h in PACU	(*n* = 18) within 1.5 h in PACU	(*n* = 7) within 2,5 h in PACU	(*n* = 5) within 4 h in PACU
21 (50)	10 (24)	200 (100–300)	1450 (501–2310)	5 (3–8)	3 (0)	50 (25–100)	5 (2–28)	9 (5–13)	6 (2–10) 5 (2–10)	3.5 (2–8) 5 (2–8)	5 (2–7) 5 (3–7)	4 (2–7) 4 (0–6)

Values are reported as median (range) for continuous values and number (percentage) for categorical variables

PCA = patient controlled analgesia; NRS = numeric rating scale

^a^NRS > 0

Perioperative compliance with the ERAS guideline elements was 81–100%. Six patients with open surgery did not receive an epidural for the following reasons: last minute decision of change in surgical approach (*n* = 1), conversion to open surgery (*n* = 2), failed catheter placement (*n* = 2), and unknown (*n* = 1).

## Discussion

We report that mobilization in the PACU starting 30 min after arriving is feasible, can be successful, and is safe for patients following elective colorectal surgery regardless of surgical approach. A structured mobilization procedure allowed us to mobilize all but one patient to sitting.

To our knowledge, this is the first study to evaluate the feasibility and safety of immediate mobilization after elective colorectal surgery. We report that it is feasible to mobilize already in PACU under the guidance of an experienced physiotherapist working with the nursing staff adding additional support and managing medical issues. Careful considerations were made to ensure patients’ safety, including that ambulating always was performed with the assistance of two caregivers. This safety policy worked well, and no serious adverse events occurred. A concern when planning this study was how much the environment and the availability of human resources and appropriate equipment (for example, walking aids, anti-slip socks) would influence the feasibility of mobilization. We found these issues to be of no concern, and the environment was not a limiting factor for mobilization.

There were some factors delaying and affecting the level of mobilization. Medical factors included symptoms of remaining sedation and pain on arrival. Insufficient pain control contributed to mobilization delay, especially 1–2 h after arrival. Hypotension, dizziness, and nausea occurred mainly within the first 2 h. Patient-related factors included unwillingness to mobilize. However, these factors did not prevent these patients from achieving their highest level of mobilization after optimizing pain control and nausea. Other limiting factors were related to the availability of staff. High workload and working schedules limited the assisting of ambulation for patients who underwent late afternoon surgery. Those factors created an intricate challenge and have to be considered in clinical practice.

The SOMS scale as a tool to structure the intervention process proved to be useful in two aspects. Firstly, its simplicity and clarity were advantageous, as it is straightforward, progressive, and intuitive. Secondly, it acted as a communication tool, which made it easy to convey the mobilization goal with the aim of increasing the patient’s motivation as well to prepare the staff. Due to the structure of mobilization, with attempts being performed regularly every half hour, aiming for an exact time of mobilization as part of the nursing protocol made it easier to prepare the patient mentally but also helped the staff to ensure good logistics.

Symptoms of pain and nausea were assessed before each allotted time of mobilization, and patients could be given medication as needed. This procedure of assessing symptoms combined with pharmacological treatment if deemed necessary, synchronized with timed mobilization attempts by a team of dedicated professionals, proved to work well in enhancing immediate mobilization. Optimizing symptoms enabled patients to reach a higher SOMS level and reduce the mobilization delay. Only one patient could not be mobilized at all during its stay in the PACU, due to insufficient pain relief despite optimization. Physical issues such as low blood pressure and dizziness were handled immediately by competent staff nearby and appropriate aids.

A recent study showed that factors such as motivation and physical tiredness influence immediate mobilization in the PACU [5]. With our approach of repeated mobilization attempts and open communication and support between patient and physiotherapist, we found that the patients remained positive and engaged to further mobilization to try to reach higher levels of mobility despite barriers.

The results of this study suggest that starting structured mobilization is possible as early as 30 min after arrival in the PACU, when it is synchronized with assessment and management of symptoms as necessary. With this approach, most patients were standing and ambulating within 2–3 h after arrival. Our results confirm those of other studies recently showing that patients could be mobilized within 2 h after abdominal surgery [19] and within 4 h after pancreas and liver surgery [20, 21]. Although we suggest that mobilization could start as soon as 30 min, the effects in patient’s outcomes still need to be investigated. Fiori’s study [22] showed that staff directed facilitation of mobilization on the day of surgery (after admission to the ward) did not improved outcomes. A recent RCT, however, has shown that mobilization started within 2 h after abdominal surgery improved respiratory peripheral and arterial oxygenation [19].

Healthy patients, with minimal comorbidities, have been discharged on the same day or the next day after laparoscopic colorectal surgery [23] and this requires very early mobilization. For increased same day pathways, a requirement would consequently seem be an immediate mobilization in the PACU since it is feasible and safe.

Previous studies have shown that most patients with open colorectal surgery have more postoperative pain compared to minimally invasive approaches [24, 25]. Our use of multi-modal analgesia while minimizing the use of opioids is likely to have contributed to immediate mobilization for our patients undergoing colorectal surgery [26]. Among the six patients undergoing open surgery without an epidural in place, we found that the pain prevented four of them from managing a higher level of mobilization before adequate pain control was achieved.

### Strengths and limitations

This was a pragmatic feasibility study in which mobilization after major surgery was stressed to its limits by initiating physical activity immediately on arrival at the PACU. The careful description of the mobilization protocol and safety routines allows replication in other settings. It could be argued that the small sample size and the lack of background population are limiting the generalizability of the results. However, the relatively uniform results regardless of variation in type of surgery, surgical approach, age, and sex suggest that this approach is adequate to initiate inclusion of more patients to test impacts on later recovery outcomes after colorectal surgery. Another limitation is the lack of patient-reported outcomes. This will be the focus in a future study.

In conclusion, this study suggests that it is feasible to initiate standardized mobilization as early as 30 min after arrival in the PACU after colorectal surgery. Forthcoming studies will address the question of whether this immediate mobilization also has a positive affect during the rest of the hospital stay and beyond.
